# The Consequences of Moral Courage in Nursing: A Narrative Inquiry

**DOI:** 10.1111/scs.70095

**Published:** 2025-08-08

**Authors:** Elina Pajakoski, Helena Leino‐Kilpi, Anto Čartolovni, Minna Stolt, Riitta Suhonen

**Affiliations:** ^1^ Department of Nursing Science University of Turku Turku Finland; ^2^ The Wellbeing Services County of Southwest Finland University Hospital Turku Finland; ^3^ Digital Healthcare Ethics Laboratory (Digi‐HeaL), School of Medicine Catholic University of Croatia Zagreb Croatia; ^4^ The Wellbeing Services County of Satakunta Pori Finland

**Keywords:** ethical conflict, ethics, holistic analysis, moral courage, narrative inquiry, nurse, qualitative study

## Abstract

**Aim:**

To holistically illustrate the consequences of nurses' morally courageous acts for patients, nurses, and work communities.

**Methodological Design and Justifications:**

Narrative inquiry was used to explore the topic in the context of encountering ethical conflicts in nursing care. Consequences of nurses' morally courageous acts were illustrated to understand the significance of the acts for the good of patients, nurses, and work communities.

**Ethical Issues and Approval:**

Ethical approval was received from the University's ethical committee. The participants were registered nurses who gave their informed consent to participate voluntarily.

**Research Methods:**

Individual in‐depth interviews with fourteen registered nurses of varying working experience were conducted in January–February 2023. Data were analysed inductively with holistic content analysis. This report follows the Consolidated criteria for reporting qualitative research (COREQ).

**Findings:**

Nurses demonstrated moral courage in ethical conflicts such as disrespectful behaviour, collaboration issues, missed care, and privacy violations. In doing so, they initiated discussions, completed written reports, admitted mistakes, and provided attentive patient care. Morally courageous acts helped mitigate the ethical conflicts, although some remained unresolved. The direct and indirect consequences were positive and negative for nurses who acted, and positive for patients and the work community.

**Study Limitations:**

The nurses considered the consequences of morally courageous acts for themselves and others, expressing their experiences, which excluded other people's perspectives. The complexity of the topic posed challenges in reporting the findings coherently, with only a few concepts.

**Conclusions:**

Nurses act morally courageously in varying ethical conflicts, indicating that they can defend morally responsible conduct. The consequences identified in this study highlight the potential for nurses who act morally courageously to promote well‐being among patients and professionals. However, complex ethical conflicts cannot always be resolved with one courageous act. Thus, healthcare organisations can aim to develop processes that facilitate nurses acting morally courageously and that enhance multi‐professional collaboration in ethical conflicts.

## 1 Background

Moral courage is an individual characteristic and a virtue of a nurse, having its roots in Aristotle's virtue ethics [1, 2, 3]. It is defined as acting morally courageously by standing up for what is considered the ethically right action in frequently emerging ethical conflicts despite potential personal negative consequences [1]. Acting morally courageously is one value‐based way to demonstrate nurses' caring for patients [2, 4] in ethical conflicts [2]. Although the risk of personal negative consequences sometimes actualises, morally courageous acts can also produce positive consequences for nurses, patients, and the work community [5, 6]. These positive consequences highlight the significance of moral courage in nursing [2, 7]. To understand the consequences and how they emerge, the preceding ethical conflicts and nurses' morally courageous acts are also illustrated in this study.

Ethical conflicts in which nurses need moral courage manifest in daily nursing care and can result from clashes in values between colleagues, coworkers, patients, or the organisation [8, 9]. These conflicts are complex and often have no perfect solution [9]. Ethical conflicts can threaten good care, patient safety, and the well‐being of nurses. For example, when a patient's death is approaching, ethical conflicts can manifest from conflicting values between professionals regarding how and when to provide palliative care [10]. Also, value clashes can emerge about adequate medication for the patients, sometimes leading to wrong or a lack of medication [8]. Patient safety can also be threatened because of the rough handling of a patient or missed care. Additionally, ethical conflicts result from poor collaboration between professionals [9]. When demonstrating their virtue of moral courage in ethical conflicts, nurses fulfil their ethical responsibility to promote health and well‐being [11, 12].

Sometimes, nurses choose to act morally courageously in ethical conflicts after conducting ethical reasoning and justifying the acts based on responsibility, professional ethics, or emotions for which the conflict has arisen [13]. Nurses' morally courageous acts show their inner strength when caring for their patients [4]. Morally courageous acts are, for example, refusing to act unethically, stopping an identified wrongdoing, and admitting their own mistakes [8]. Initiating a discussion about a conflict with a manager [14], completing a notice within one's organisation, and informing someone outside the organisation, such as a trade union representative or policy‐makers, are also considered morally courageous acts [14]. Furthermore, nurses act morally courageously by doing their best in work [1], respecting patients' views and needs [2], and being truly present for a patient despite the risk of personal negative consequences [2].

Consequences of nurses' morally courageous acts, standing up for what is right, can be beneficial for the patients they care for, their work community, and nurses themselves. Positive and negative consequences resulting from nurses' morally courageous acts have been identified for nurses and patients. For morally courageous nurses, positive consequences include the development of competence [15], increased respect in society [14, 15, 16], a boost in confidence to act morally courageously [16], developed resilience [17] and increased well‐being [18]. Furthermore, managers and colleagues may show appreciation [6, 14, 16]. However, morally courageous acts can trigger negative consequences for nurses [14, 16]. These possible negative consequences include bullying [6], discrimination from colleagues or managers [6], loss of a job [1, 6] and being blamed for acting selfishly [15]. Nevertheless, the potential good outweighs the risk of personal negative consequences, highlighting the importance of moral courage [1, 2]. Positive consequences for patients include psychological support [15], increased safety [18], prevented poor practice, and good care [16]. For the work community, positive consequences, such as encouragement to act morally courageously [16], a positive work environment, and salvaging a potentially unethical situation, have been reported [18].

Research on nurses' morally courageous acts in ethical conflicts has described general positive consequences for patients and nurses and negative consequences for morally courageous nurses. There is a gap in research regarding comprehensive descriptions comprising the ethical conflicts nurses face, the morally courageous acts they choose to perform despite personal risks, and the consequences of the acts for the nurses, patients, and work communities.

This study presents moral courage as a virtue of nurses, the perspective having been used and recommended in earlier research [2, 7]. As moral courage itself cannot be seen, the exploration focuses on nurses' descriptions of their morally courageous acts and the consequences [7]. As a justification for the study, this exploration contributes to understanding the consequences of nurses' morally courageous acts as part of the complex entirety comprising ethical conflicts, morally courageous acts, and consequences, and how all these are interconnected. This can highlight the significance of nurses' moral courage and the good achieved from it.

## 2 Aim and Research Questions

This study aimed to holistically illustrate the consequences of nurses' morally courageous acts. To reach this aim, ethical conflicts, morally courageous acts, and the resulting consequences are illustrated as experienced and described by nurses. The research question is:

What are nurses' morally courageous acts in ethical conflicts, and the consequences of the acts for the ethical conflict, the acting nurse, the patient, and the work community?

## 3 Methods

### 3.1 Research Design

A narrative inquiry with a holistic content analysis was used as it facilitates a comprehensive exploration of a topic [19]. This report follows the Consolidated criteria for reporting qualitative research (COREQ) [20].

### 3.2 Sampling and Context

Purposive sampling was used to reach registered nurses with experience in ethical conflicts, who acted morally courageously despite the risk of personal negative outcomes and identified the consequences of their morally courageous acts [21]. An invitation to participate and information about the study were published in the national nursing‐themed online discussion forum hoitajat.net from December 2022 to February 2023. The discussion forum is freely accessible [22]. The inclusion criteria for participation were that the participant was a registered nurse who had experienced ethical conflicts and had acted morally courageously despite the risk of personal negative outcomes. The potential participants determined whether they had the required experience of ethical conflicts and acting morally courageously and contacted the first author by phone or email.

#### 3.2.1 Participants

Fourteen registered nurses with work experience in somatic, mental health, and substance abuse care in inpatient hospital wards and outpatient clinics in Finland participated. Their working experience in nursing varied from 7 to over 30 years.

#### 3.2.2 Data Collection

The data were collected during January–February 2023, with individual in‐depth interviews [19] carried out face‐to‐face or through Zoom [23]. The data were gathered for two studies: the current report and another article [13]. The interview guide was piloted among two healthcare professionals. No changes were made after the pilot. In the interview, participants were asked to talk about two types of situations: ethical conflicts requiring moral courage, in which they had not acted morally courageously, as well as times when they had responded to ethical conflicts by acting courageously. The participants described the ethical conflicts they had faced, their morally courageous acts, and the consequences of those acts. The interviewer (first author) asked probing questions to lead the discussion along the studied topics in the interview guide. The interviews took ~25–90 min, with only the interviewer and participant present. The interviews were audio recorded and transcribed with the participants' consent. After each interview, field notes were made to describe the atmosphere and non‐verbal communication. Data collection continued until theoretical saturation.

### 3.3 Data Analysis

The data were analysed inductively using holistic content analysis to holistically illustrate the findings [19]. The first author was responsible for conducting the analysis, which started with thoroughly reading each transcribed interview to gain a comprehensive view of them. The corresponding field notes were used to revisit the atmosphere of each interview. From each interview, the ethical conflicts, the morally courageous acts, and the consequences of the act for the ethical conflict, the acting nurse, the patient, and the work community were identified.

Next, the ethical conflicts, the morally courageous acts, and the consequences of the acts from all interviews were merged, and the analysis continued focusing on the merged contents. The findings of the analysis were presented as holistic illustrations comprising the nurses' descriptions of ethical conflicts, their morally courageous acts, and the ensuing consequences. To understand the consequences, three perspectives for presenting them were identified: to whom the positive or negative consequence was related, whether the conflict was resolved or remained unresolved, and whether the consequences resulted directly from the morally courageous act or if other actions were conducted; the consequence ensued indirectly after those [19].

## 4 Ethical Considerations

Good scientific practice was followed [24]. Ethical approval was received from the Ethics Committee of the University of Turku on 24.10.2022. The participants received information about the study and the first author's contact details before voluntarily participating, giving written informed consent before the interview [24]. Personal data, including age, length of work experience in health care (years), current and previous fields of nursing, and work roles, were processed following the EU General Data Protection Regulation (2016/679) [25].

## 5 Findings

### 5.1 Ethical Conflicts and Nurses' Morally Courageous Acts

The nurses described ethical conflicts in which they acted morally courageously. The conflicts comprised problems in collaboration, disrespectful behaviour, violation of privacy, and missed or wrong care (Table 1). Ethical conflicts will be presented as part of holistic illustrations together with the morally courageous acts and the consequences of the acts.

**TABLE 1:** Ethical conflicts in which nurses acted morally courageously despite the risk of personal negative consequences.

The ethical conflict	Description of the ethical conflict
Problems in collaboration between professionals	Unequal dividing of work tasks between individuals or professional groups
	Unwillingness to collaborate with other professional groups
	Insufficient competence of professionals
Disrespectful behaviour	Another professional acting disrespectfully towards the nurse
	Another professional acting disrespectfully towards the patient
Violation of privacy	Patient's privacy is violated
	Nurse's privacy is violated
Missed or wrong care	Lack of or wrong medication due to conflicting values of professionals
	Lack of or wrong care plan due to conflicting values of professionals
	Lack of necessary patient examinations due to conflicting values of professionals
	Ignorance of the patient's religion or spirituality in the care plan
	Violation of the patient's autonomy

Nurses acted morally courageously for the good of the patient and nurses' well‐being at work. The acts included initiating discussion either in the situation or after it, with a person involved or another professional, such as a nurse leader or a physician, and filing notices inside or outside the organisation. Moreover, the nurses refused to do tasks unrelated to patient care, to be truly present with the patients. They also courageously admitted when they made mistakes (Table 2).

**TABLE 2:** Nurses' morally courageous acts.

Morally courageous act	Description of the act
Initiating discussion	Discussion with another professional in the situation
	Discussion with a leader or someone else after the situation
Written notice	Filling a notice inside the organisation
	Filling a notice outside the organisation
Being truly present with the patient	Refusing to do tasks unrelated to patient care.
	Having more time for the patient
Admitting one's own mistake	Telling others about their errors, e.g., concerning medication

### 5.2 Direct and Indirect Consequences of Nurses' Morally Courageous Acts

Direct consequences ensued straight from the morally courageous act, while indirect consequences manifested after additional actions were conducted based on the morally courageous acts. Table 3 shows the direct and indirect consequences according to the person to whom the consequence is related and whether the ethical conflict was resolved. Similar direct positive consequences manifested in varying situations, such as a nurse feeling pleased for the sake of the patient and getting support from other professionals. Correspondingly, indirect negative consequences that were similar in varying situations included, for example, a nurse being discouraged due to being treated badly by others. Additionally, there were positive consequences directly related to the ethical conflict, one example being when an ethical conflict was related to poor collaboration, and the collaboration between professionals therefore became smoother (Table 3).

**TABLE 3:** Direct and indirect consequences of nurse's morally courageous acts.

Consequences of nurses' morally courageous acts				
	Regarding the ethical conflict	Regarding the nurse	Regarding the patient	Regarding the work community
The conflict was resolved	*Solving the conflict through collaboration* Re‐arranging patient care and nurses' work^a^	*Being pleased* ^a^ Being pleased for the sake of the patient Being relieved that the conflict was resolved	*Actualisation of rights* Getting the correct medication^a^ Getting the correct treatment in the correct unit^a^ Getting better circumstances at the time of death^b^	*Improved collaboration* ^b^ Collaboration got smoother Work tasks were divided equally
	Development of professionals' competence or skills^b^	*Being empowered* ^a^ Feeling that work is meaningful Strengthened courage	*Mental wellbeing* Getting care that acknowledges spirituality^b^ Getting time to say goodbye to family before death^a^	*Improved competence* ^b^ Clinical competence Moral competence
				*Improved atmosphere* ^b^ Ethical safety Feeling of togetherness
The conflict remained unresolved	Other solutions were required^b^ The ethical conflict was prolonged^b^ More problems were identified^b^	*Being discouraged* ^b^ Feeling bad for the sake of the patient Worsened mental wellbeing Moral distress	Missed care^b^	
General consequences regardless of if the conflict was resolved or remained unresolved		*Support from other professionals* ^a^ Getting support from colleagues, co‐workers, and leaders Getting thanks from colleagues, co‐workers, leaders, and patients		
		*Mixed feelings* ^a^ Acting courageously was a tough process		
		*Being treated badly by others* ^a^ Being blamed, called names, or laughed at Being the target of others getting angry		

^a^ Direct consequence.

^b^ Indirect consequence.

#### 5.2.1 Holistic Illustrations of the Consequences of Morally Courageous Acts Regarding the Resolution of Ethical Conflicts

Directly or indirectly, nurses' morally courageous acts led to some ethical conflicts being resolved while others remained unresolved. A direct consequence was when a conflict was resolved through collaboration with colleagues or other professionals. This collaboration, including discussions and meetings, also led to indirect positive consequences: a common understanding of the conflict between professionals or between the nurse and the patient.

The following example depicts an instance of missed care due to inadequate medication as well as a clash between a physician's values and the patient's right to good care and medication. The nurse identified a violation of the physician's ethical responsibility to provide adequate medication and demonstrated their own caring for the patient and responsibility with the morally courageous act. The act was initiating discussion despite the risk of angering the physician, which had happened before. The indirect positive consequence was that collaborative meetings were arranged.

We've had a problem with [a physician in the ward] for years […] the physician wouldn't like to prescribe opiates for patients in any situation […] they did not want to prescribe opiates because if they were in that situation themselves, they wouldn't want to have their head confused […] this problem was quite… quite big in the ward and, it was important to me, as it was my area of responsibility. […] So, I contacted the physician responsible for pain medication in the hospital and told them about the matter […] Well, that physician had talked to the other physician but they couldn't do more… and the situation did not improve. Next, a meeting was arranged; there was the physician responsible for pain medication, and our physician, and me, and the nurse leader, and another nurse responsible for pain medication. […] the situation [meeting] then proceeded, in the end, so that we talked it through that, you know, how they [the physician] should act and why. (Participant 2)

Resolving the ethical conflict with a morally courageous act was not always possible, and sometimes the conflict continued or grew. The nurses then had to come up with other solutions to try to make the situation better, an indirect consequence being that further ethical conflicts were identified after the morally courageous act. The following example involves another professional acting disrespectfully towards the patient.

[Another professional] met a patient and was, you know, back facing the patient and… and spoke over the patient, seemingly asked the patient's view but then in the end, did not even listen, did not care at all, did not show empathy […] I went, after working there for 2 weeks, to speak to the nurse leader, you know, to tell about these matters, and the leader once again already knew. […] It was very discouraging, when… you know… the leader just shrugs… I got a strange feeling that… how is it possible that the leader does not intervene or how is it possible that the leader doesn't take the matter further [to solve it]? (Participant 9)

In the example above, the patient seemed to feel bad about the disrespectful acting. Thus, the nurse courageously talked to the leader about the unethical conduct of the other professional. However, the leader did not do anything, and the situation stayed the same, illustrating an indirect negative consequence.

#### 5.2.2 Holistic Illustrations of Ethical Conflicts, Morally Courageous Acts, and Consequences of the Acts for Nurses, Patients, and the Work Community

**FIGURE 1:**
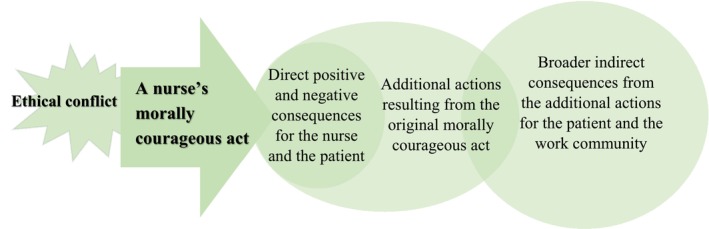
Direct and indirect consequences of nurses' morally courageous acts for various stakeholders.

The nurses reported direct and indirect consequences of their morally courageous acts for themselves, patients, and their work communities (Figure 1). Additionally, morally courageous acts served as a starting point for improving or resolving ethical conflicts and often led to additional actions. These actions could have wider indirect positive consequences for patients and the work community (Table 3, Figure 1).

##### 5.2.2.1 Consequences for the Morally Courageous Nurse

The nurses experienced positive and negative consequences due to their morally courageous acts, consequences that manifested either when the conflict was resolved or stayed unresolved, sometimes in both cases. Direct positive consequences included feeling pleased and empowered and receiving support and thankfulness from colleagues, co‐workers, and leaders.

The example below describes an ethical conflict regarding a missing care plan for a palliative patient, who was suffering. The morally courageous act was to contact another professional, and the direct positive consequences for the nurse were being pleased for the patient's sake and feeling empowered (Figure 1, Table 3).

There was a patient in palliative care, who was actively suffering, the symptoms were taken care of, but in a way […] the situation was left hanging, no‐one had the courage to decide […] if it was a situation with terminal care. […] Then we had… one really… wonderful anaesthesiologist who devotedly liked to take care of these palliative care patients […] and our understanding of ethical care was similar. […] I couldn't come up with anything else than to phone that person. […] And then the care plan was clear and we got the appropriate palliative care. […] I feel that … you know, my work effort was truly meaningful, that I could really make things better and from those experiences, I have more courage for the next problematic situation. (Participant 5)

Sometimes nurses described indirect negative consequences for themselves, illustrating the risk of possible negative outcomes being actualised. The negative consequences included being discouraged, worsened mental well‐being, and being treated badly by others. Acting morally courageously was described as a tough experience for nurses: they felt bad for the patients and had mixed feelings when conflicts went unresolved. Also, nurses relayed situations when they left their work due to unresolved ethical conflicts. Some had experienced being treated badly by others, including being blamed, bullied, or laughed at. Additionally, nurses told of other professionals or the patient's next of kin getting angry with the nurse.

Some nurses tried to do as little work as possible, and they tried to work as fast as possible, without thinking about how good the quality of care they were providing was […] I tried to, you know, to find a positive way to talk to these colleagues […] how to find ways why we should provide… patient‐centred care, and not just fast care, but you know, holistic care for the patient's well‐being. […] Then, the colleagues laughed at me behind my back. (Participant 6)

In the example above, other nurses were avoiding work tasks and working hurriedly without caring about the quality or ethics of care. The morally courageous act was to initiate discussion with the colleagues involved and the indirect negative consequence was being laughed at by others (Figure 1, Table 3).

##### 5.2.2.2 Consequences for the Patient

The positive consequences that the nurses identified demonstrated times when their aim to do good for the patient was actualised. The consequences resulted directly from a morally courageous act or indirectly after other actions ensued from the courageous act (Figure 1). The direct consequences for the patients included the actualisation of rights and improved mental well‐being, which manifested when the ethical conflict was resolved. Actualising of rights could mean receiving good care, correct medication, correct examinations, correctly timed care or, as an indirect consequence, good circumstances at the time of death (Table 3).

The following example involved a nurse admitting their own mistake.

[After a nightshift] I got a feeling that […] I had diluted the antibiotics incorrectly… and I came home and… I phoned right away […] and I said to the other colleague to please check that… I just got a feeling that I had done it incorrectly, and that they have to be redone. And she went [to check] and said ‘How did you know?’ […] and I would have been sorry if I hadn’t, you know, done everything for the patient. […] and… the mistake was corrected, and you know, the patients got the correct medicine.(Participant 14)

The following example involved a nurse admitting their own mistake. “[After a nightshift] I got a feeling that […] I had diluted the antibiotics incorrectly… and I came home and… I phoned right away […] and I said to the other colleague to please check that… I just got a feeling that I had done it incorrectly, and that they have to be redone. And she went [to check] and said ‘How did you know?’ […] and I would have been sorry if I hadn't, you know, done everything for the patient. […] and… the mistake was corrected, and you know, the patients got the correct medicine” (Participant 14).

In the example above, the nurse realised after their work shift that they had made a mistake. The nurse courageously admitted the mistake. As a direct consequence, a colleague corrected the mistake, the indirect consequence was the patients getting the correct medication (Figure 1, Table 3).

##### 5.2.2.3 Consequences for the Work Community

Nurses described how consequences sometimes ensued indirectly from additional actions, such as discussions and improvements being conducted because of a morally courageous act (Figure 1). The identified indirect consequences for the work community were all positive: improved collaboration, competence of professionals, and a better atmosphere. Improved collaboration included equal sharing of work tasks and continuous follow‐ups on collaboration. Improved competence comprised clinical and moral competence. The nurses felt that the ethical climate became safer, that there was a feeling of togetherness, and other professionals supported their views and values. Sometimes the ethical conflicts and morally courageous acts were related to problems in multiprofessional collaboration, and the consequences of the acts were beneficial for the whole work community.

A problem concerning multi‐professional collaboration and teamwork. […] There was a new teamwork procedure in the ward, and it had led to these kinds of side effects, that people formed teams that were too tight […] so there was this team of practical nurses who felt that they didn't need the registered nurses' work at all. […] one registered nurse came to say to me that they had tried to tell [the team] that there is medication for a patient, that should have been going on for days, and regular check‐ups… but the message didn't go through and there hadn't been a registered nurse on the team for days. […] Then I decided to arrange a meeting and a discussion about it… that what does that mean when they don't accept registered nurses… two professional groups do not collaborate… you know, it was a big meeting […] and I think I managed to change things. […] So it was very important and people were thankful, and I think the things changed [for the better]. (Participant 13)

In the example above, an ethical conflict manifested as problems in collaboration between professional groups in organising and sharing work tasks in the unit. One nurse courageously initiated a meeting, which promoted solving the problem, and the collaboration improved. An indirect consequence ensued after conducting other actions based on the morally courageous act (Figure 1, Table 3).

## 6 Discussion

The findings of this study present holistic illustrations of the ethical conflicts, morally courageous acts that nurses carry out in the conflicts, and the consequences of those acts. The holistic illustrations provide a new, wide perspective for understanding this complex entirety. Nurses demonstrate caring for their patients and doing good for the work community with their morally courageous acts. Previous research has described direct consequences ensuing from nurses' morally courageous acts for the acting nurse and the patient [14, 15, 17, 18]. However, in addition to direct consequences, this study provided new knowledge of indirect positive consequences related to additional actions ensuing from morally courageous acts, illustrating the broader implications of nurses' moral courage. The additional actions, such as facilitating collaboration and education, promote the well‐being and competence of the morally courageous nurse and the work community, as well as good care for patients [7]. The findings illustrate that nurses' morally courageous acts can enhance the good of patients, nurses, and work communities and promote the quality of care. The morally courageous acts did not have direct negative consequences. However, sometimes the nurses experienced negative consequences from other people. Thus, morally courageous acts can be seen as a positive way for nurses to resolve ethical conflicts despite personal risks, as the acts promote the good of the morally courageous nurse, patients, and the work community [2].

The nurses aimed to resolve ethical conflicts by acting morally courageously despite possible personal negative outcomes [2]; corresponding with earlier research [8]. The ethical conflicts reflect contemporary nursing, for example, with an insufficient number of nurses or other resources related to missed care [26] as well as challenges in collaboration [8]. The nurses felt that acting morally courageously was worth taking personal risks, and they described knowingly taking risks in favour of what was right based on their values [1]. This illustrates moral courage as a specific area of the nurses' ethical conduct; while the risk is not always present in general ethical conduct, nurses always take personal risks when acting morally courageously [1, 2, 3]. Taking personal risks in favour of patients' good out of love and caring demonstrates nurses' virtue of moral courage [4]. The morally courageous acts of initiating discussion, filing a written notice, being truly present with the patient, and admitting their mistakes correspond with earlier research [2, 6, 8, 27]. However, in this study, the acts are presented holistically together with the ethical conflicts and consequences, illustrating the significance of nurses' moral courage in promoting ethical patient care, a global goal of nursing [11, 28].

As for the consequences, it is worth acknowledging from the perspective of virtue ethics that nurses decided to act morally courageously based on their values. Thus, the nurses were virtuous and acted morally courageously according to what was right, not based on possible consequences. They identified the consequences later when holistically describing the ethical conflicts, morally courageous acts and the ensuing consequences in the interviews. The positive direct and indirect consequences of nurses' morally courageous acts highlight the significance of nurses showing moral courage in their work [29]. Ethical conflicts were either resolved or remained unresolved after a morally courageous act, an observation supported by research regarding whistleblowing [6]. However, even if a nurse aims to resolve an ethical conflict, it is not always possible to resolve complex issues with only one act. Thus, it is important for the sake of ethical quality of care to acknowledge the complexity of ethical conflicts [9] and to facilitate collaboration between the professionals involved [30].

Positive consequences for the acting nurse empower the nurse to act morally courageously in future ethical conflicts [31]. Additionally, feeling pleased after resolving an ethical conflict emphasises the significance of nurses' moral courage—good for the patients means good for themselves [16]. Patients benefit from morally courageous acts when a conflict is resolved, even if they do not observe the acts, highlighting the importance of nurses' moral courage for well‐being in society [29]. The nurses conveyed that acting morally courageously is worth the risk of personal negative consequences [1, 32], as they were pleased with their acts. This illustrates the core of moral courage: taking personal risks while aiming for the good of someone else [1, 2].

When nurses aim to do good for others, there are wider indirect consequences for the work community. The morally courageous acts were starting points for conducting larger‐scale improvements, such as providing education to develop competence and making care processes smoother. This finding highlights the crucial role of nurses' moral courage in care teams, as nurses work close to the patients and are in a key position to defend good, value‐based care [33]. Thus, as an implication for practice, maintaining and promoting moral courage among nurses [17] remains important for the sake of promoting ethical conduct in nursing care [33]. Moreover, it is important to acknowledge in healthcare organisations that resolving ethical conflicts is a collective responsibility. The work community can identify processes for resolving ethical conflicts after they have been courageously brought into discussion. The processes can involve shared governance, conducted in collaboration with ethics committees and organisation management, including mentoring programmes [33].

### 6.1 Strengths and Limitations

This study produced knowledge of the direct and broad indirect consequences that nurses' morally courageous acts can have on ethical conflicts, nurses, patients, and work communities, adding to the field of nursing ethics. As a strength, three of the authors have a background in nursing and all authors have expertise in nursing ethics research, which supports an understanding of the participants' role as moral actors in nursing. The purposeful sample of nurses with experience of moral courage was appropriate for a narrative inquiry, as the premise for understanding the study topic was in the experience‐based knowledge of the participants. The narratives provided rich and informative data, strengthening the credibility of the study [19].

The study also has strengths and limitations in terms of *width*, *coherence*, *insightfulness*, and *parsimony* [19]. The *width* of the findings is seen in the holistic illustrations and the quotations. The holistic analysis provides an understanding of the ethical conflicts, morally courageous acts, and their consequences. As a limitation regarding the *width*, the nurses reflected on their experiences and the consequences to other people; thus, the timeline of events in their narratives can be ambiguous, and other perspectives are not represented. Nevertheless, the aim was to understand the consequences from the perspective of the morally courageous nurses. Furthermore, the aim was to reach *coherence* by illustrating ethical conflicts, morally courageous acts, and their consequences together as a meaningful entirety. However, the complexity of the topic posed challenges to presenting the findings coherently. Additionally, following the analysis frame systematically contributed to the credibility of the findings. The broader significance of the consequences regarding the work community illustrates the *insightfulness* of the findings. Finally, as a limitation, the complexity of the findings posed challenges to reaching *parsimony*, although the aim was to present the findings with logical, holistic illustrations [19].

### 6.2 Implications for Further Research

Further research could identify ways to develop and facilitate multi‐professional collaboration in ethical conflicts. This could help nurses avoid severe negative consequences for acting morally courageously and thus enhance morally courageous acts as part of ethical conduct. Also, processes and organisational structures supporting moral courage among nurses could be developed in collaboration with healthcare organisations. These topics could be studied with narrative, survey, and action‐research designs to reach comprehensive findings that can be smoothly implemented in organisations.

## 7 Conclusions

Nurses' value‐based morally courageous acts in ethical conflicts show that they can demonstrate the virtue of moral courage by caring for their patients as well as defending morally responsible conduct. The direct and particularly the broad indirect consequences highlight the potential for morally courageous nurses to promote the good and well‐being of not only the patients but also the professionals. Furthermore, it is important to acknowledge that ethical conflicts, due to their complexity, often cannot be resolved with one courageous act. Thus, it is worth acknowledging in healthcare organisations that collaboration among professionals in resolving ethical conflicts is essential. Based on these findings, healthcare organisations can aim to develop processes to enhance moral courage among nurses and processes to deal with the identified conflicting situations, which could facilitate improvements in the organisations.

## Author Contributions

E.P., R.S., and H.L.‐K. planned the study; R.S. and H.L.‐K. are doctoral researcher's (EP) supervisors. E.P., R.S., and H.L.‐K. obtained ethics approval. E.P. conducted the interviews and transcribed the data. E.P. analysed the data with support and collaboration from R.S. and H.L.‐K. E.P. prepared the full draft of the article. R.S., H.L.‐K., M.S., and A.Č. provided theoretical and substantial expertise and input. All authors contributed to and approved the final version of the manuscript.

## Ethics Statement

Ethical approval was received from the University and informed consent was obtained from all participants.

## Conflicts of Interest

The authors declare no conflicts of interest.

## Data Availability

The authors have nothing to report.
